# Highly comparable metabarcoding results from MGI-Tech and Illumina sequencing platforms

**DOI:** 10.7717/peerj.12254

**Published:** 2021-09-30

**Authors:** Sten Anslan, Vladimir Mikryukov, Kęstutis Armolaitis, Jelena Ankuda, Dagnija Lazdina, Kristaps Makovskis, Lars Vesterdal, Inger Kappel Schmidt, Leho Tedersoo

**Affiliations:** 1 Institute of Ecology and Earth Sciences, University of Tartu, Tartu, Tartumaa, Estonia; 2 Mycology and Microbiology Center, University of Tartu, Tartu, Tartumaa, Estonia; 3 Department of Ecology, Institute of Forestry of Lithuanian Research Centre for Agriculture and Forestry (LAMMC), Kaunas, Lithuania; 4 Latvian State Forest Research Institute SILAVA, Riga, Latvia; 5 Department of Geosciences and Natural Resource Management, University of Copenhagen, Copenhagen, Denmark

**Keywords:** Metabarcoding, COI, Illumina, NovaSeq, DNBSEQ, MGI-Tech

## Abstract

With the developments in DNA nanoball sequencing technologies and the emergence of new platforms, there is an increasing interest in their performance in comparison with the widely used sequencing-by-synthesis methods. Here, we test the consistency of metabarcoding results from DNBSEQ-G400RS (DNA nanoball sequencing platform by MGI-Tech) and NovaSeq 6000 (sequencing-by-synthesis platform by Illumina) platforms using technical replicates of DNA libraries that consist of COI gene amplicons from 120 soil DNA samples. By subjecting raw sequencing data from both platforms to a uniform bioinformatics processing, we found that the proportion of high-quality reads passing through the filtering steps was similar in both datasets. Per-sample operational taxonomic unit (OTU) and amplicon sequence variant (ASV) richness patterns were highly correlated, but sequencing data from DNBSEQ-G400RS harbored a higher number of OTUs. This may be related to the lower dominance of most common OTUs in DNBSEQ data set (thus revealing higher richness by detecting rare taxa) and/or to a lower effective read quality leading to generation of spurious OTUs. However, there was no statistical difference in the ASV and post-clustered ASV richness between platforms, suggesting that additional denoising step in the ASV workflow had effectively removed the ‘noisy’ reads. Both OTU-based and ASV-based composition were strongly correlated between the sequencing platforms, with essentially interchangeable results. Therefore, we conclude that DNBSEQ-G400RS and NovaSeq 6000 are both equally efficient high-throughput sequencing platforms to be utilized in studies aiming to apply the metabarcoding approach, but the main benefit of the former is related to lower sequencing cost.

## Introduction

Metabarcoding, the identification of organisms *via* DNA marker genes from environmental samples or a mixture of heterospecific specimens (Taberlet et al., 2018), is a powerful tool in biodiversity analysis (Kelly et al., 2018; Pont et al., 2021; Valentin et al., 2019; Watts et al., 2019). This approach has been efficiently used to characterize the community composition of microbial and animal taxa from various types of environmental samples such as soil (Bahram et al., 2018; Nilsson et al., 2019), water (Djurhuus et al., 2018; Liu et al., 2020), sediments (Kang et al., 2021; Wurzbacher et al., 2017), dust (de Groot et al., 2021; Rocchi et al., 2017) and feces (Ando et al., 2020; Anslan et al., 2021). In animals, metabarcoding has also been widely used to identify host-associated microbiomes, determine the structure of entire holobionts and dietary differences in various species (Alberdi et al., 2019; Kueneman et al., 2019). The information acquired through DNA marker gene sequencing has greatly boosted our knowledge about the ecology and distribution patterns of various aquatic and terrestrial animal groups such as nematodes, arthropods and annelids (Arribas et al., 2016; Beng & Corlett, 2020; Compson et al., 2020; Deiner et al., 2017; Zawierucha et al., 2021).

Since the mid-2000s, the metabarcoding technique has greatly benefited from technological advances in library preparation, primer and sample-specific index design, novel sequencing platforms as well as from optimized bioinformatics workflows and accumulating reference data (Taberlet et al., 2018; Nilsson et al., 2019). Short-read, second-generation high-throughput sequencing (HTS) technologies are currently the most widely used means for metabarcoding due to a relatively low cost per sample, high sequencing depth and accuracy. Sequencing instruments produced by Illumina, Inc. (*e.g*., MiSeq and NovaSeq) using sequencing-by-synthesis technology are dominating the market as they offer viable solutions for both ultra-high sequencing depth and paired-end sequencing of short- and mid-sized amplicons (up to 500–600 bases; Kumar, Cowley & Davis, 2019). By utilizing recent advances in DNA nanoball sequencing technology (Drmanac et al., 2010; Li et al., 2019), MGI-Tech, Inc. has produced several DNBSEQ (MGISEQ) platforms with similar throughput and quality profiles compared with Illumina sequencing (Jeon et al., 2021; Kumar, Cowley & Davis, 2019). The results from Illumina and MGI-Tech sequencing platforms are highly comparable and may be used interchangeably for RNA sequencing and whole genome sequencing (Jeon et al., 2019; Kim et al., 2021; Korostin et al., 2020). However, the error rate of DNBSEQ technology (MGI-2000 instrument) was marginally higher than for Illumina (HiSeq instrument) when using 2 × 150 paired-end sequencing mode on both platforms (quality scores >30: 95.03% and 97.18% for MGISEQ-2000 and HiSeq 2500, respectively; Korostin et al., 2020). The results of these early genome sequencing-oriented studies suggest that MGI-Tech platforms may be used efficiently also in metabarcoding studies. In early 2021, sequencing costs for MGI-Tech DNBSEQ-T7 were about 50% lower compared with Illumina NovaSeq platform (cost per read) for the greatest throughput analyses (Tedersoo et al., 2021). So far, only a single metabarcoding study has been conducted to compare these sequencing platforms (DNBSEQ-G400 and Illumina MiSeq) for recovering rRNA gene 16S and ITS amplicons of bacterial and fungal mock communities (Sun et al., 2021). For the ITS2 amplicon, Sun et al. (2021) reported small but significant differences between DNBSEQ-G400 and MiSeq platforms, but this difference can be attributed to use of different primer pairs for DNA library preparation.

Here, we aim to compare the relative performance of DNBSEQ-G400RS (2 × 200 bp) and Illumina NovaSeq 6000 (2 × 250 bp) for DNA metabarcoding. DNA libraries were prepared from the same pools of mitochondrial cytochrome oxidase 1 (COI) amplicons generated from soil DNA extracts.

## Methods

### Sampling

We selected 120 sites (Table S1) in an area of 500 km^2^ around Tartu, Estonia, which included various terrestrial ecosystems (managed croplands, abandoned croplands, plantations of fruit trees and forestry trees on former agricultural land and old forests (>80 years)). In each site, we established a sampling plot (30 × 30 m) in a homogeneous patch to minimize any edge effects and vegetation gradient effects. In a 3 × 3 grid, we collected nine soil cores from each plot, with coordinates representing the central location (Table S1). Soil cores for analyses of soil bulk density were collected by hammering a PVC tube (50 mm diam.) to 100 mm depth after removing loose litter. Using a clean knife (sterilized in 1% NaOCl solution), roughly 15 g of soil was scraped from sides of the same core holes and pooled into a clean Zip-Lock plastic bag. The composite sample was well mixed and frozen immediately among freezing tablets (initial temperature, −86 °C). In the laboratory, the frozen samples were transferred to −80 °C.

### Molecular analyses

The frozen samples were crushed using a hammer in double plastic bags (to speed up thawing), placed into paper bags and dried in a drying cabinet at 35 °C for 24 h. The dried samples were transferred into Zip-Lock bags, followed by initial homogenisation by vigorous rubbing by hands. Roughly 1 g of homogenised soil dust was transferred into an Eppendorf tube and subjected to further homogenisation by using two 3-mm steel balls at 30 Hz. Altogether 0.25 g of soil powder was subjected to DNA extraction using a Thermo Scientific KingFisher Flex robot and MagAttract PowerSoil kit (Qiagen Inc., Hilden, Germany), following the manufacturer’s instructions.

For amplification *via* PCR, primers mlCOIintF (5′ GGW ACW GGW TGA ACW GTW TAY CCY CC; Leray et al., 2013) and jgHCO2198 (3′ TAI ACY TCI GGR TGI CCR AAR AAY CA; Geller et al., 2013) were used to target the ~313 bp mitochondrial cytochrome oxidase 1 (COI) gene. The former primer was barcoded with unique phase shift indexes (Table S2). The 25 µl PCR mixture comprised five µl of 5× HOT FIREPol Blend Master Mix (Solis Biodyne, Tartu, Estonia), 0.5 µl of each forward and reverse primer (20 mM), one µl of DNA extract and 18 µl ddH_2_O. Thermal cycling included an initial denaturation at 95 °C for 15 min; 25 cycles of denaturation for 30 s at 95 °C, annealing for 30 s at 57 °C, elongation for 1 min at 72 °C; final elongation at 72 °C for 10 min and storage at 4 °C. All PCR reactions were performed in duplicate and pooled for subsequent analyses. PCR products (five µl) were verified using 1% agarose gel electrophoresis. Samples yielding no product were re-amplified with 30 cycles, followed by pooling and gel electrophoresis. Both a negative control (ddH_2_O with no DNA template) and a positive control sample (an artificial DNA molecule with multiple primer sites) were used to assess obvious contamination during sample preparation for PCR and the efficiency of PCR, respectively. The PCR products were normalized for preparation of two libraries based on visual inspection of band strength on an 1% agarose gel. We used the following criteria: no band (*i.e*., negative control) = 10 µl; faint band = seven µl; medium band = three µl; strong band = one µl. The pooled amplicons were shipped for Illumina NovaSeq 6000 (2 × 250 bp; hereafter NovaSeq) paired-end sequencing in Novogen Inc., UK and for MGI-Tech DNBSEQ-G400RS (2 × 200 bp; hereafter DNBSEQ) paired-end sequencing in Clinomics Inc., South Korea. The service providers were selected strictly based on the best price offer for delivering 50 million reads (Table 1). Sequencing libraries for respective sequencing platforms (including adapter ligation) were prepared by the service providers from the same amplicon pool. NEBNext® Ultra™ II DNA Library Prep Kit (PCR-free workflow) was used for NovaSeq (at Novogene Inc., Cambridge, UK) and MGIEasy FS DNA Library Prep Set (includes PCR step after ligation) was used for DNBSEQ library preparation (at Clinomics Inc., Ulsan, South Korea). The sequencing costs are provided in Table 1.

**Table 1:** Cost calculations for Illumina NovaSeq 6000 and MGI-Tech DNBSEQ-G400RS based on the best offering service providers and data retrieved (euros).

	NovaSeq 6000 (2 × 250 bp)	DNBSEQ-G400RS* (2 × 200 bp)
Library preparation for sequencing	100	170
Offer for sequencing 50 million reads	1,000	170
Actual cost per million raw reads	30.07	7.21
Actual cost per million filtered reads (matrix#1)	53.23	11.92
Actual cost per raw gigabit (Gb)	26.44	8.25
Actual cost per filtered Gb (merged and quality filtered)	104.76	33.66

**Note:**

* Originally in USD; converted to EUR as of 2021-05-10 (invoice issued).

### Bioinformatics

NovaSeq and DNBSEQ provided 42,990,088 and 55,581,045 raw reads, respectively. The raw reads were demultiplexed using cutadapt v3.4 (Martin, 2011) by requiring full-length index coverage (–overlap 12) and allowing one mismatch to index sequence (−e 1) but no indels (–no-indels) (Data 1). Fasta-formatted index file specifying sample-index combinations served as an input for cutadapt. The reads were separated into sample-wise files according to this information. During demultiplexing, we also accounted for reverse complementary sequences in the raw data by running two rounds of demultiplexing using cutadapt. For the second round, the unassigned R1 and R2 reads from the first round used as inputs. Demultiplexed reads from both runs were then merged by sample. Prior to further processing, all sequences were reoriented to 5′–3′ orientation based on the PCR primers. For this procedure, fqgrep (v0.4.4; Das, 2011) was used by allowing two mismatches to primer sequences as implemented in PipeCraft v1.0 (Anslan et al., 2017). Paired-end sequences were assembled using vsearch v2.17.0 (Rognes et al., 2016) with the following settings: –fastq_minovlen 10, –fastq_minmergelen 10, –fastq_maxdiffs 20, –fastq_maxns 0, –fastq_maxmergelen 600, –fastq_allowmergestagger. Both forward and reverse primers were trimmed from the sequences using cutadapt by allowing two mismatches to primer strings and primer match overlap of 24 but no indels. Reads where both primers remained undetected were discarded. Quality-filtering of the remaining sequences was performed using trimmomatic v0.39 (Bolger, Lohse & Usadel, 2014) with the following options: SLIDINGWINDOW:5:30, LEADING 11, TRAILING 11. Putative chimeric sequences were removed using the *uchime_denovo* algorithm as implemented in vsearch (–id 0.97 for pre-clustering, default options for chimera detection). All filtered sequences from both sequencing platforms per sample were merged and clustered into operational taxonomic units (OTUs) with 97% sequence similarity threshold using vsearch (–cluster_size –iddef 2). During the latter process, a uniform sample-by-OTU table (containing data from both platforms) was generated (matrix#1, see below). The resulting OTUs were classified using BLAST+ v2.10.1 (Camacho et al., 2009) against the CO1Classifier database v4 (Porter & Hajibabaei, 2018) (Data 2).

In addition to the OTU workflow, amplicon sequence variants (ASVs) were calculated using DADA2 (v1.18; Callahan et al., 2016). The ASV matrix was generated including a subset of 60 samples from the total of 120 samples that were used for generating OTU matrices (Table S1). The quality filtering options included removal of all sequences with ambiguous bases (maxN = 0), trimming low quality ends (truncQ = 2) and keeping sequences with maximum expected error rates of one (maxEE = 1). Chimeras were removed with the ‘consensus’ method. All other processes, including denoising, followed the default DADA2 workflow (Data 1). Inputs for the ASV pipeline included fastq files for each sample that comprised primer-trimmed and reoriented reads (as specified above). The ASVs were further subjected to post-clustering at 97% sequence similarity threshold using the LULU algorithm (Frøslev et al., 2017) to merge consistently co-occurring AS*Vs*. Thus, two sets of ASVs matrices were generated for the analyses: (1) ASVs matrix, and (2) post-clustered ASVs matrix (Data 3).

### OTU data matrices

Four types of sample-by-OTU data matrices were prepared (Data 1) to compare the DNBSEQ and NovaSeq sequencing platforms: (1) matrix#1—all ‘raw’ OTUs as outputted after the clustering step; (2) matrix#2—‘raw’ OTUs but global singletons (*i.e*., OTUs that had only one sequence across matrix#1) removed; (3) matrix#3—only metazoan global non-singleton OTUs; (4) matrix#4—rarefied metazoan OTUs. To account for variations in sequencing depth, metazoan OTUs data (matrix#3) was rarefied using phyloseq v1.34.0 (McMurdie & Holmes, 2013) to a depth of 10,489 reads per sample (matrix#4). To reduce the remaining putative artefacts in the matrix#3, OTUs with a representative sequence length different from the expected amplicon length (313 bp ± 4 bp) were discarded. This eliminated 59% (82,036) of OTUs accounting for 31.8% (15,639,811) of reads from matrix#2. Besides Metazoa, the used COI primers amplified a wide variety of other non-target eukaryotes (mostly fungi) as well as prokaryotes. An OTU was assigned to Metazoa (in matrix#3) when the best blastn match of the query sequence had at least 90% query coverage and 75% identity against the reference sequence. For metazoan taxonomic group statistics, an OTU received a phylum level classification when the best blastn match of the query sequence (an OTU) had ≥80% identity against the reference sequence with phylum-level annotation. Some OTUs were best matched to Hydrozoa and Porifera at <89% sequence similarity, but since these aquatic organisms are unexpected in terrestrial environments, we assigned these OTUs to unclassified Metazoa.

### Statistics

Differences in the OTU/ASV richness between DNBSEQ and NovaSeq data sets were tested using paired *t*-tests in STATISTICA (v7; StatSoft Inc., Tulsa, OK, USA). For ASV matrices and OTU matrices#1–3, we first calculated the predicted richness values based on residuals of OTU richness, as derived from linear regression analyses using natural logarithm transformed sequencing depth as an independent variable, separately for DNBSEQ and NovaSeq data subsets. For the OTU matrix#4, residuals were not calculated because of using rarefaction. Spearman correlation was used to examine the sequencing depth and OTU richness correlations between sequencing platforms. Mantel tests (with 9999 permutations, method = ‘spear’), as implemented in the ‘vegan’ package v2.5.7 (Oksanen et al., 2015) in R v4.0.4 (R-Core-Team, 2021), were used to test correlations between corresponding sample similarities from different sequencing platforms. Additionally, Procrustes tests (with 9999 permutations, metaMDS ordination), as implemented in the ‘vegan’ package, were used to compare correlation in community structure as revealed from DNBSEQ and NovaSeq instruments. Bray–Curtis similarity of Hellinger-transformed data were used for both Mantel and Procrustes tests. To assess OTU/ASV overlap between sequencing platforms, Venn diagrams were drawn using Venny 2.1 (Oliveros, 2018). The proportion of potential index-switching errors was estimated using the UNCROSS2 score (Edgar, 2018) with default parameter values (*f* = 0.01, *t*_min_ = 0.1) for each sample and sequencing platform combination. Differences among sequencing platforms were tested using a Bayesian generalized linear mixed model with binomial errors and logit link, where ‘sample’ was used as a random effect. The model was fitted with Stan v2.21 (Stan-Development-Team, 2021) and brms package v2.15.0 (Bürkner, 2017) using seven Markov chains of Hamiltonian Monte Carlo, with 15,000 sampling iterations and 2,000 warm-up iterations for each chain.

## Results

**Figure 1:**
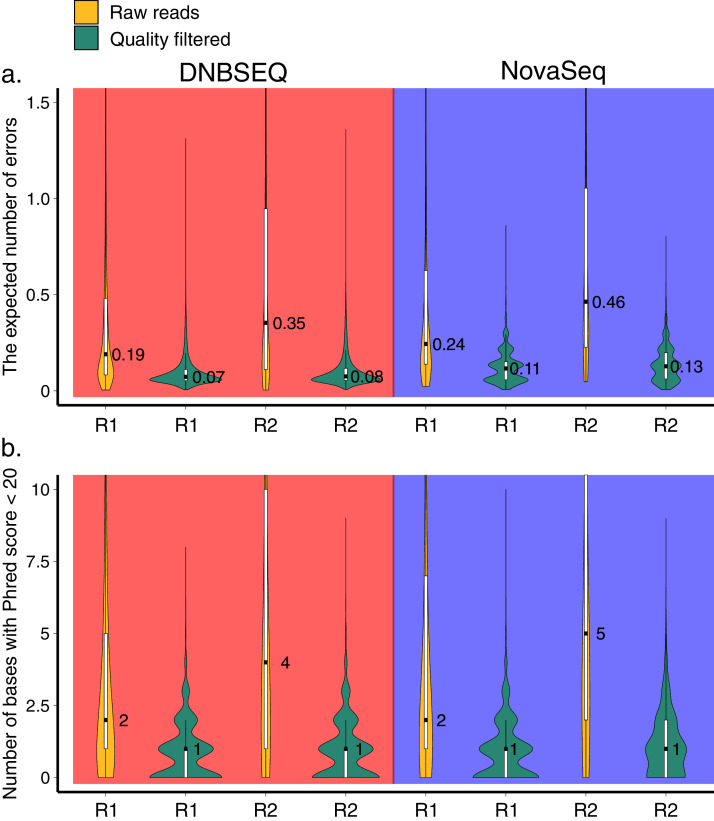
Quality profile. Distributions of the expected number of errors (A) and the number of bases with Phred score <20 (B) per read in raw (yellow) and trimmomatic quality-trimmed (green) reads obtained with DNBSEQ and NovaSeq platforms for a single sample (G5300; 392,379 and 270,651 raw reads in DNBSEQ and NovaSeq data, respectively). Median, quartiles, and the 1.5 interquartile range are shown on the boxplots inside the violin plots. Due to the highly skewed distributions, *y*-axes were truncated to 1.5 (A) and 10 (B). The expected number of errors per sequence was estimated following Edgar & Flyvbjerg (2015).

Demultiplexed HTS datasets of 120 samples from DNBSEQ and NovaSeq contained 50,129,600 and 39,813,707 sequences, respectively. The overall quality score distributions exhibited similar profiles between DNBSEQ and NovaSeq datasets (Fig. 1; Fig. S1). However, the latter exhibited marginally higher level of expected number of errors in the sequences (Fig. 1A). Therefore, after filtering (all filtering steps before clustering in the OTU workflow), proportionally more sequences were discarded in the NovaSeq data (48.1%) compared with DNBSEQ data (43.1%; Table S3). Similarly, after the ASVs workflow (for the subset of 60 samples out of 120), the average proportional sequence loss per sample was higher in NovaSeq data (29.5% *vs*. 33.4%; Table S3).

**Figure 2:**
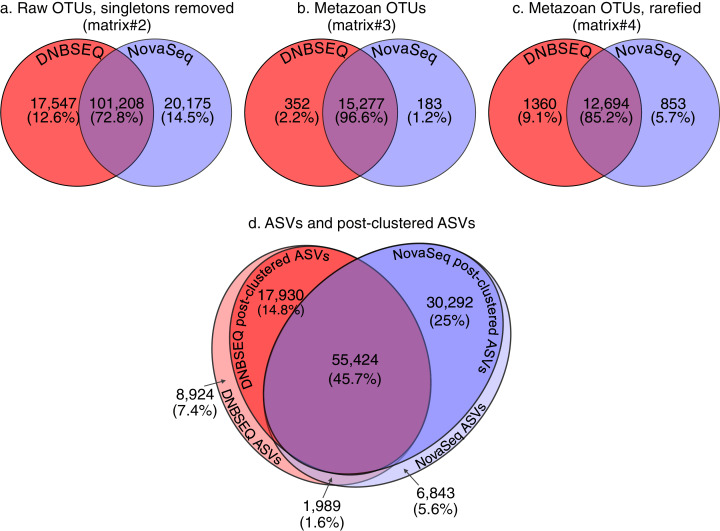
Venn diagrams. Venn diagrams demonstrating the number and proportions of shared and unique OTUs per sequencing platform: (A) raw OTUs, global singletons excluded (matrix#2); (B) metazoan OTUs, global singletons excluded (matrix#3); (C) rarefied metazoan OTUs (matrix#4); (D) ASVs and post-clustered ASVs matrices (note that ASVs data is for a subset of 60 samples). The total proportion of shared ASVs is 47.3%, but 53.5% when considering only post-clustered ASVs.

Clustering at 97% sequence similarity threshold (both datasets merged) revealed 182,066 OTUs including 43,136 singletons. Of the 138,930 non-singleton OTUs, 17,547 (12.6%) were unique to DNBSEQ and 20,175 (14.5%) unique to NovaSeq (Fig. 2A). These unique OTUs usually comprised a low number of reads, with a median sequence count of 3 (±13.7 SD) and 5 (±61.1 SD) for DNBSEQ and NovaSeq data, respectively. The proportion of shared OTUs between datasets was 72.8% for all non-singleton OTUs (matrix#2), 96.6% for the full metazoan dataset (matrix#3) and 85.2% for the rarefied metazoan dataset (matrix#4; Fig. 2). Total number of ASVs (in a subset of 60 samples, across both data sets) was 121,402, including 2,660 global singletons. Post-clustering those ASVs with 97% sequence similarity threshold with LULU algorithm merged 17,756 ASVs (14.6%), retaining 103,646 ASVs (including global 343 singletons). The proportion of shared ASVs between post-clustered ASVs data set was 53.5% (Fig. 2D). The unique post-clustered ASVs per platform had median sequence count of 7 (±56.9 SD) and 5 (±42.0 SD) for DNBSEQ and NovaSeq data, respectively.

### Taxonomic composition

**Figure 3:**
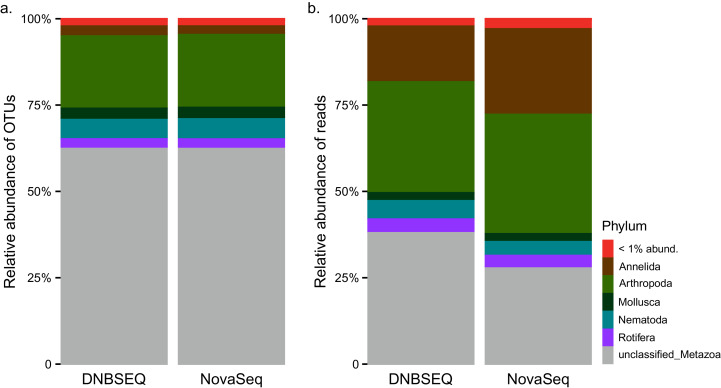
Taxonomy. Phylum-level bar plots indicating proportional distribution of metazoan OTUs (A) and sequences (B) from DNABSEQ and NovaSeq platforms (data matrix#3).

Metazoa contributed to 13.2% and 12.7% OTU richness, and 18.6% and 24.4% total read abundance, in the DNBSEQ and NovaSeq datasets, respectively. Within Metazoa, the largest phyla in terms of OTU richness were Arthropoda and Nematoda, whereas the largest classes were Insecta (Arthropoda) and Chromadorea (Nematoda) (Fig. 3). While the distribution of relative OTU numbers was highly similar across sequencing platforms, there were certain differences in relative abundance of reads. In the DNBSEQ data, relatively more reads of unclassified Metazoa were recovered at the expense of Annelida (Fig. 3). Taxonomic annotation of ASVs were not performed in this study.

### Sequencing depth and diversity

**Figure 4:**
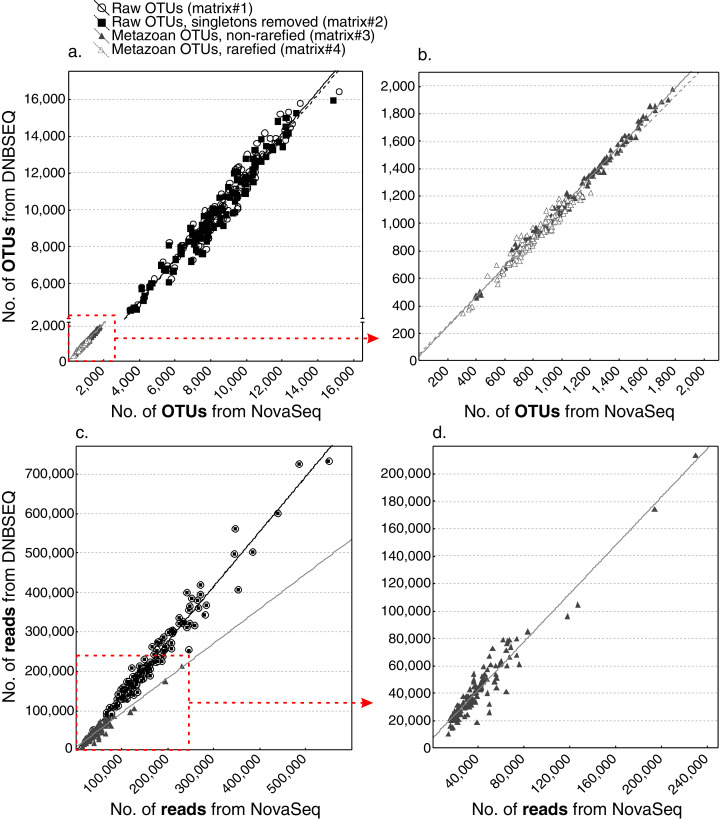
Richness correlations. Correlation of OTU richness (A and B) and read abundance (C and D) per sample as provided by DNBSEQ and NovaSeq platforms (Spearman *R* ≥ 0.91, *N* = 120, *P* < 0.001 for all matrices using logarithm-transformed data). Plot b. represents a zoom-in for OTU matrix#3 and #4. Plot d. is a zoom-in for matrix#3. Note that the differences of number of reads from matrix#1 and matrix#2 are relatively low, thus the points completely overlap in the low-resolution plot c.

**Figure 5:**
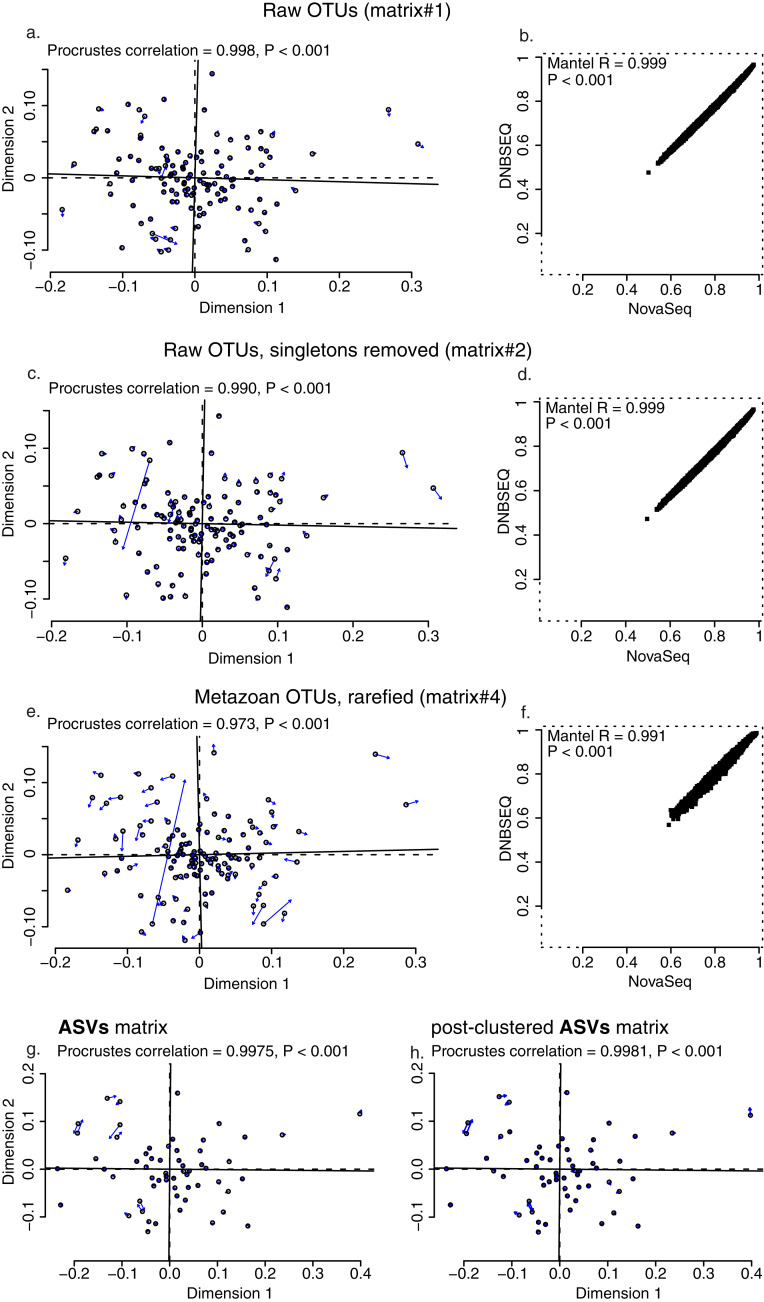
Community correlations. Procrustes (A, C and E) and Mantel test plots (B, D and F) for OTU data matrices: (A and B) raw OTUs (matrix#1), (C and D) raw OTUs without singletons (matrix#2), (E and F) metazoan OTUs with rarefied data (matrix#4). Plots for metazoan global non-singleton OTU matrix (matrix#3) not displayed here, but the analyses revealed the same results (significantly high correlation between data sets, Procrustes correlation = 0.995, *P* < 0.001; Mantel *R* = 0.996, *P* < 0.001). Open circles in the Procrustes plots denote ordination configuration of NovaSeq data and arrows point to the configuration in DNBSEQ data ordination.

While the total sequencing depth among platforms depended on the sequencing amount ordered and cannot be thus compared, the relative proportion of reads per sample was highly similar across the two platforms (min: 0.18% and 0.18%; max: 2.57% and 2.66%; median: 0.73% and 0.73%; average: 0.83% and 0.83% for DNBSEQ and NovaSeq, respectively; Fig. S2). All four OTU matrices exhibited strong correlations in per-sample OTU richness between sequencing platforms (Fig. 4A), with Spearman correlation coefficients 0.974, 0.974, 0.994 and 0.970 for raw OTUs (matrix#1), non-singleton OTUs (matrix#2), metazoan OTUs (matrix#3) and rarefied metazoan OTUs (matrix#4), respectively (*P* < 0.001 for all tests). Similarly, all OTU matrices exhibited strong correlations in per-sample sequence abundance between sequencing platforms (Fig. 4B), with Spearman correlation coefficients 0.975, 0.975 and 0.912 for matrix#1, matrix#2 and matrix#3, respectively (*P* < 0.001 for all cases). In addition, community composition retrieved by DNBSEQ and NovaSeq platforms were strongly correlated based on Procrustes (*R* ≥ 0.97 and *P* < 0.001 for all tests) and Mantel (mantel *R* ≥ 0.991 and *P* < 0.001 for all tests) statistics (Fig. 5). These patterns were the nearly identical when comparing ASV matrices of DNBSEQ and NovaSeq (Fig. 5; Fig. S3).

**Figure 6:**
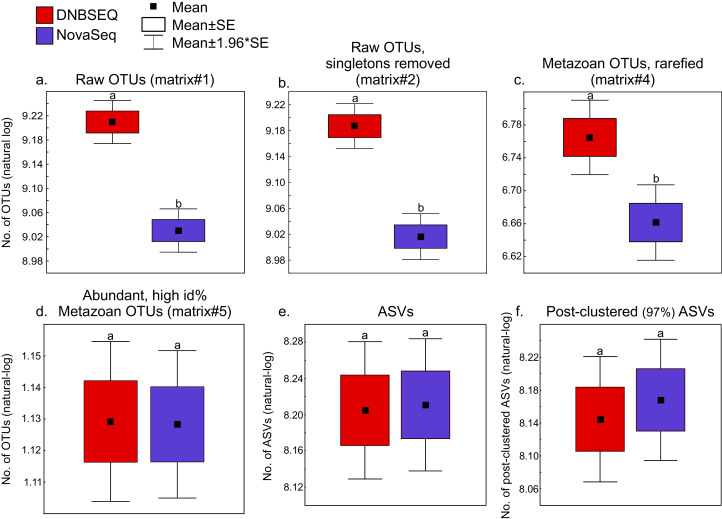
Richness. Box-plots showing platform-wise differences in OTU/ASV richness in (A) raw OTUs (matrix#1), (B) raw OTUs without singletons (matrix#2) and (C) metazoan OTUs (rarefied; matrix#4) richness data. Box-plot for metazoan global non-singleton OTU matrix (matrix#3) not displayed here, but the analyses revealed the same results (significantly higher number of OTUs in the DNBSEQ data set, *P* < 0.001). (D) number of ASVs and (E) post-clustered ASVs. The ASVs dataset includes a subset of 60 samples out of total 120 samples (note that OTU results were the same for corresponding 60 samples as reported here for the 120 samples). Different letters above the whiskers denote statistical differences of the means.

**Figure 7:**
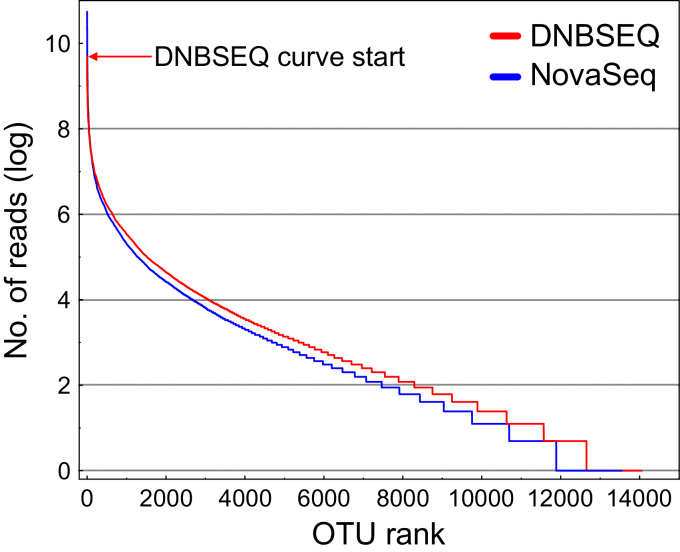
Rank abundance curves (RAC). Rank abundance curves for rarefied metazoan OTUs (matrix#4) based on natural logarithm transformed abundance. The top position of the DNBSEQ curve is marked with a red arrow. The most abundant DNBSEQ OTU ranks seventh overall. The six most abundant NovaSeq OTUs are on average 2.3-fold more abundant than the corresponding DNBSEQ OTUs.

There was a significant difference in OTU richness between DNBSEQ and NovaSeq data in all OTU matrices (matrix#1: *t* = 39.191, df = 119, *P* < 0.001; matrix#2: *t* = 40.140, df = 119, *P* < 0.001; matrix#3: *t* = 15.755, df = 119, *P* < 0.001; and matrix#4: *t* = 22.723, df = 119, *P* < 0.001) (Figs. 6A–6C). For example, an average per-sample OTU richness was 9.7% higher in DNBSEQ data in the rarefied metazoan dataset (matrix#4). The rank abundance curves of OTUs (matrix#4) derived from DNBSEQ and NovaSeq displayed a slightly different pattern (Fig. 7). There was a slight tendency towards greater dominance in the NovaSeq dataset, with the top three abundant OTUs being more abundant by a factor of 2.9, 3.2 and 1.9. We further explored the differences in OTU richness between data sets by removing all potential ‘noise’ of spurious OTUs by further filtering matrix#4 (rarefied metazoan OTU table) to include only OTUs with relative sequence abundance of ≥0.01% (per data set) and ≥98% sequence similarity to the reference sequences (matrix#5 in Data 1). In this stringently filtered data set, differences in OTU richness disappeared (paired *t*-test: *t* = 0.131, df = 119, *P* = 0.896; Fig. 6D). Similarly, there were no significant differences in the ASV richness between DNBSEQ and NovaSeq data sets in the ASV matrices (*P* > 0.9; Figs. 6E and 6F).

### Index-switching errors

**Figure 8:**
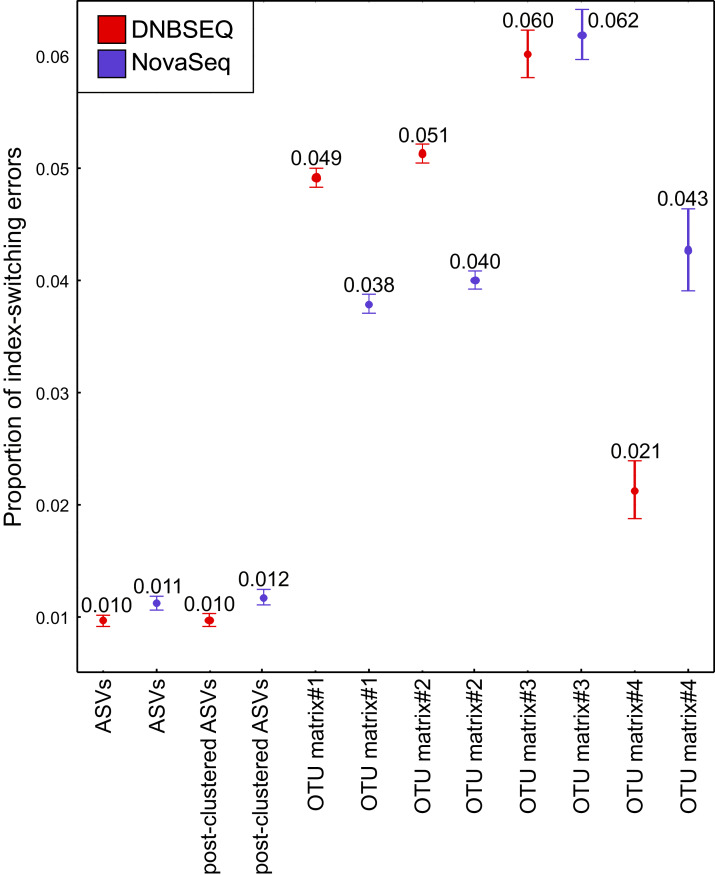
Index switches. The proportion of index-switching reads for tested OTU and ASV matrices from DNBSEQ (red) and NovaSeq (blue) sequencing platforms. Error bars denote 95% confidence intervals (see Table S5). *N* = 120 for OTU matrices and *N* = 60 for ASV matrices. The index-switching proportions were similar as displayed here when testing the OTU tables comprising 60 corresponding samples.

The UNCROSS2 score revealed that index-switching errors were slightly higher in the DNBSEQ OTU matrices #1 and #2 (Fig. 8; Table S5). For example, the overall proportion of reads that represent putative index switches were 0.049% and 0.038% for DNBSEQ and NovaSeq data in matrix#1, respectively (Fig. 8). However, in the rarefied metazoan dataset (matrix#4), the DNBSEQ matrix displayed a lower proportion of index-switching errors compared with the NovaSeq data (0.021% *vs*. 0.043%; Fig. 8; Table S5). This indicates that rarefaction either lowers the detection of index-switching errors or the majority of index-switches (which occur in low abundances) were removed during the process. However, compared with the OTU matrices, the ASV matrices displayed a relatively lower proportion of reads with putative index-switching errors, and the data from both platforms exhibited similar level of index switches (Fig. 8). Procrustes correlations between index-switch corrected and uncorrected OTU tables were high (0.977–0.999; *P* < 0.001; Fig. S3), indicating that quantitative community-level analyses are weakly impacted by these low proportions of index-switching errors.

## Discussion

### Recovering OTU/ASV richness and composition

By using the same amplicon pools of ~313-bp COI marker gene fragment for platform-specific library preparation and sequence data generation on DNBSEQ-G400RS and NovaSeq 6000 platforms, we demonstrate strongly correlating community and richness profiles. The overall similarities between two short-read sequencing platforms corroborate earlier studies on metabarcoding of bacteria (Sun et al., 2021) and genomics of various organisms (Jeon et al., 2021; Kim et al., 2021).

The OTU and ASV community patterns showed strong correlations between the sequencing platforms (Fig. 5; Fig. S3), but the DNBSEQ dataset revealed on average 9.7% higher OTU richness per sample (rarefied metazoan OTUs, matrix#4). This may be related to a lower effective read quality leading to generation of spurious OTUs (Edgar 2017). To test this, we clustered the unique NovaSeq and DNBSEQ metazoan OTUs in matrix#4 at 96% sequence similarity. Altogether 10.2% and 12.9% of the unique NovaSeq and DNBSEQ OTUs (in matrix#4) clustered to other OTUs using this relaxed threshold. This difference suggests that the greater number of closely related OTUs may result from a slightly higher proportion of remaining erroneous reads in the DNBSEQ data. Furthermore, the DNBSEQ data exhibited lower relative abundance (in terms of number of reads) of the most common OTUs compared with the NovaSeq dataset (Fig. 7), which may also result from a higher proportion of sequencing errors. However, if this greater dominance is artefactual, occupation of a large proportion of sequences by the dominants may render rare species undetected and result in a lower overall richness (Elbrecht, Peinert & Leese, 2017). Therefore, less biased sequencing depth towards high abundant taxa may result in overall greater detected richness (Elbrecht, Peinert & Leese, 2017). In this study, we did not include a relevant mock community and therefore, we cannot compare whether the results of one or the other platform are closer to the reality in terms of Metazoan diversity. However, the indications about the higher proportion of remaining erroneous reads in the DNBSEQ data (after quality filtering) was also supported by the disappearance of significant differences in the OTU richness when comparing the stringently filtered matrix#5 (Fig. 6D). Furthermore, the ASV matrices demonstrated highly similar richness profiles between different platforms (Figs. 6E and 6F). The ASV workflow included the DADA2 denoising algorithm (Callahan et al., 2016), which seems to efficiently remove the remaining ‘noise’ and resulting in highly concurrent ASV and post-clustered ASV richness profiles between the sequencing platforms (Figs. 6E and 6F). A majority of the ‘noise’ is ‘hidden’ in the molecular units (OTUs or ASVs) with low read count, especially in the sample-wise singletons. Compared with the full ASV matrix (both DNBSEQ and NovaSeq data), the sample-wise singletons were 65 times more abundant in the OTU matrix#2 (a comparison across 60 samples; Data 1), which contributed to the OTU richness differences between sequencing platforms. The bioinformatics workflow with the additional denoising step lowers the fraction of low-abundance spurious molecular units, which inflate the richness (Reitmeier et al., 2021). Additionally, rare OTUs (*i.e*., OTUs with low number of reads) are poorly reproducible between sequencing runs (Leray & Knowlton, 2017). Therefore, non-stringent quality-filtering may increase richness heterogeneity for the same samples sequenced in different runs (Reitmeier et al., 2021). Despite the differences in OTU richness in our study, the OTU community level analyses from either platform would yield highly corresponding results (as indicated by the high Procrustes and Mantel correlations, >0.97; Fig. 5); however, an additional denoising and filtering low abundant molecular units may aid towards more accurate richness analyses.

### Index switches

Potential index-switching errors in the raw OTU matrices #1 and #2 were slightly higher in the DNBSEQ than NovaSeq data (Fig. 8; Table S5). This may be at least partly related to the library preparation processes (by service providers) prior to sequencing. The NovaSeq library preparation included a PCR-free workflow, whereas the DNBSEQ library was subjected to post-ligation PCR which may have significant effect on index switching (Schnell, Bohmann & Gilbert, 2015; Caroe & Bohmann, 2020). While index switches had a negligible impact on the community analyses, such a slightly higher index switch rate in the DNBSEQ data may partly explain the observed differences in per-sample OTU richness (Fig. 6). Following rarefaction (matrix#4), the proportion of potential index-switching errors decreased considerably. This indicates that many potential index-switching errors were removed by discarding a large proportion of sample-wise rare OTUs (with low read abundance), which are more likely to be technical artefacts. Because of higher sequencing depth in the DNBSEQ data in our study (and higher per-sample singleton OTU proportion in matrix#3), latter data set lost proportionally more reads and probably therefore the proportion of putative index switches declined slightly more in the DNBSEQ data (Fig. 8). Because many low abundant sequences (especially singletons) were removed during the ASV workflow, the index switches in the corresponding matrices displayed markedly lower proportion of putative index-switching errors, with a highly similar proportion of index switches remaining in both datasets (Fig. 8). Although index switches are a known issue in high-throughput sequencing platforms (Carlsen et al., 2012; Caroe & Bohmann, 2020; Loit et al., 2019; Schnell, Bohmann & Gilbert, 2015), we found that it had a minor effect on the community structure in our tested datasets (Fig. S5). Nonetheless, being aware of the presence of such errors and applying appropriate data curation prior to statistical analyses are principal requisites of a scientific study (Caroe & Bohmann, 2020; Esling, Lejzerowicz & Pawlowski, 2015).

### Limitations

The main limitation of this study is no replication of sequencing runs with companies providing a similar service. However, the single runs of DNBSEQ-G400RS and NovaSeq 6000 revealed similar results, which is unlikely to occur when one or both of these runs have technical issues or biases in library preparation. By testing the reproducibility of 16S amplicon sequencing results from Illumina MiSeq platform, Wen et al. (2017) demonstrated that the OTU community variations were greater between technical replicates that were subjected to different sequencing runs compared with variations that were derived from technical replicates within the same sequencing run. Relatively higher variations between different sequencing runs are likely arising because of the low reproducibility of rare OTUs (*i.e*., OTUs with low number of reads; Leray & Knowlton, 2017). Here, we intentionally excluded a mock community because we did not access various axenically grown animals.

## Conclusions

We demonstrate that the MGI-Tech DNBSEQ-G400RS and Illumina NovaSeq 6000 instruments are both well suited for DNA metabarcoding of COI amplicon libraries of ~313 bases given the similarities in data quality and reconstruction of animal diversity. However, we caution that amplicon length (beyond 350 bases) and length heterogeneity (some amplicons beyond 350 bases such as in fungal Internal Transcribed Spacer, ITS) may become critical for the 2 × 200 paired-end chemistry of the MGI-Tech DNBSEQ-G400RS instrument. We conclude that the main benefit of DNA nanoball sequencing lies in its lower sequencing costs (Table 1).

## Supplemental Information

10.7717/peerj.12254/supp-1 Supplemental Information 1 Supplemental Figures.

10.7717/peerj.12254/supp-2 Supplemental Information 2 Sampling plots.

10.7717/peerj.12254/supp-3 Supplemental Information 3 Used PCR primers for COI amplicon library construction. Only forward primer was indexed. For sequencing, platform specific adapters were ligated by the sequencing

10.7717/peerj.12254/supp-4 Supplemental Information 4 Track reads. Number of sequences per sample after each bioinformatic process.

10.7717/peerj.12254/supp-5 Supplemental Information 5 Quality score estimates for a sample G5300. Data appears in Figure 1.

10.7717/peerj.12254/supp-6 Supplemental Information 6 The proportion of reads and OTUs with index switch errors for tested OTU table types from DNBSEQ and NovaSeq sequencing platforms. ’index-switch %’ data appears in Figure 8.

10.7717/peerj.12254/supp-7 Supplemental Information 7 OTU matrices and codes.

10.7717/peerj.12254/supp-8 Supplemental Information 8 OTU blastn annotations.

10.7717/peerj.12254/supp-9 Supplemental Information 9 ASV matrices.

## Additional Information and Declarations

### Data Availability

The following information was supplied regarding data availability: The raw sequencing data is available in the Sequence Read Archive: PRJNA743174. All data matrices generated from the sequencing data and used for the analyses are available in the Supplemental File.
