# Supplementary material for: Highly comparable metabarcoding results from MGI-Tech and Illumina sequencing platforms
Source: PeerJ. 2021 Sep 30;9:e12254. doi: 10.7717/peerj.12254 (PMC8491618; doi:10.7717/peerj.12254)
Supplement: Supplemental Information 1 [file peerj-09-12254-s001.pdf]

Highly comparable metabarcoding results from MGI-Tech and Illumina sequencing platforms

Sten Anslan, Vladimir Mikryukov, Kęstutis Armolaitis, Jelena Ankuda, Dagnija Lazdina, Kristaps Makovskis, Lars Vesterdal, Inger Kappel Schmidt, Leho Tedersoo

Supplementary Figures:

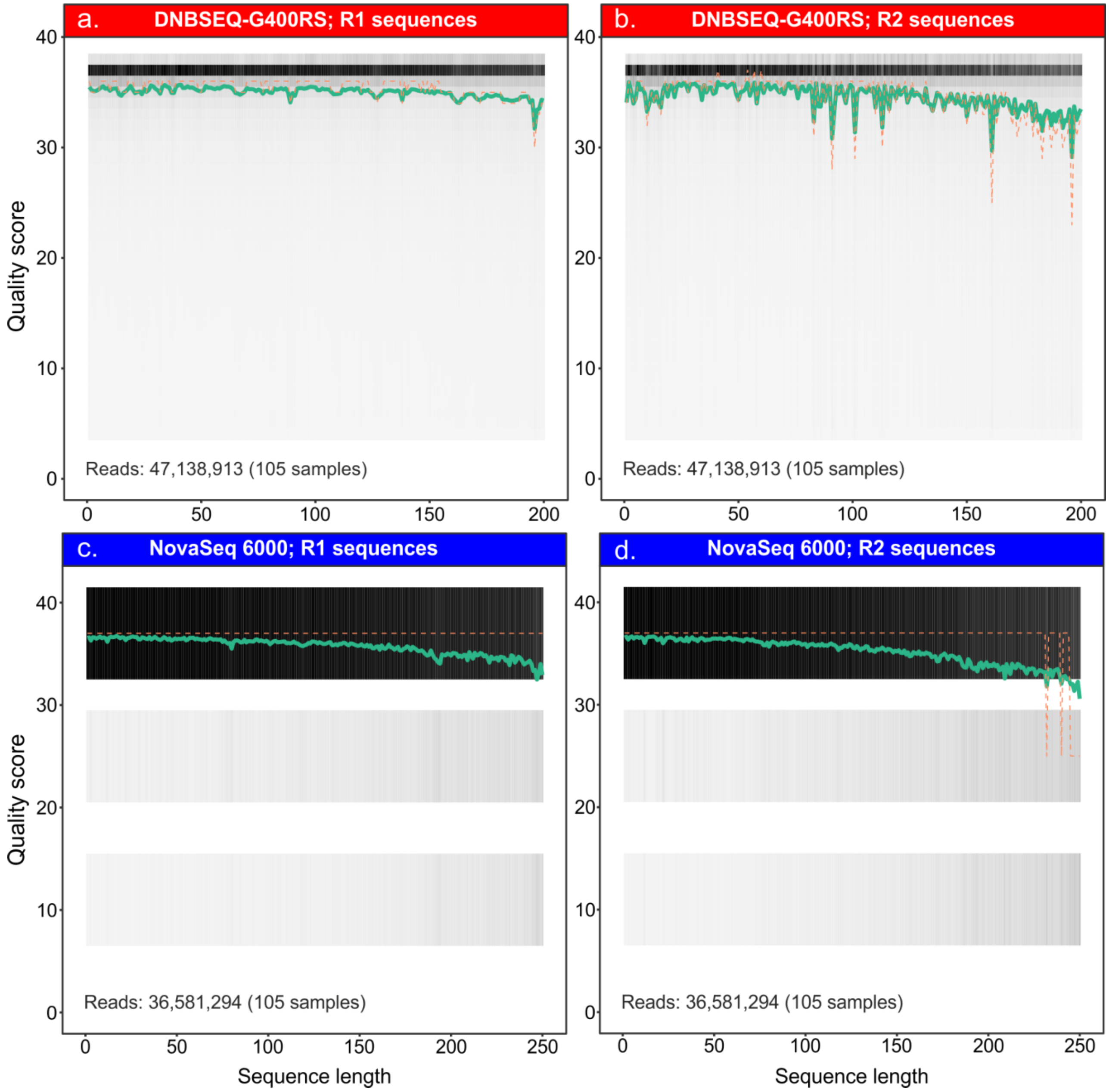

Figure S1. Sequence quality scores profiles for DNBSEQ (a., b.) and NovaSeq (c., d.) data (pooled subset of 105 samples out of 120 samples; generated with 'plotQualityProfile' function of DADA2). Green line denotes the median quality score, and orange dotted line denotes the quartiles of the quality score distribution. Gray-scale heat map denotes the frequency of each quality score at each base position (note that NovaSeq data has binned quality scores (2, 11, 25, 37), therefore the distinct heat map).

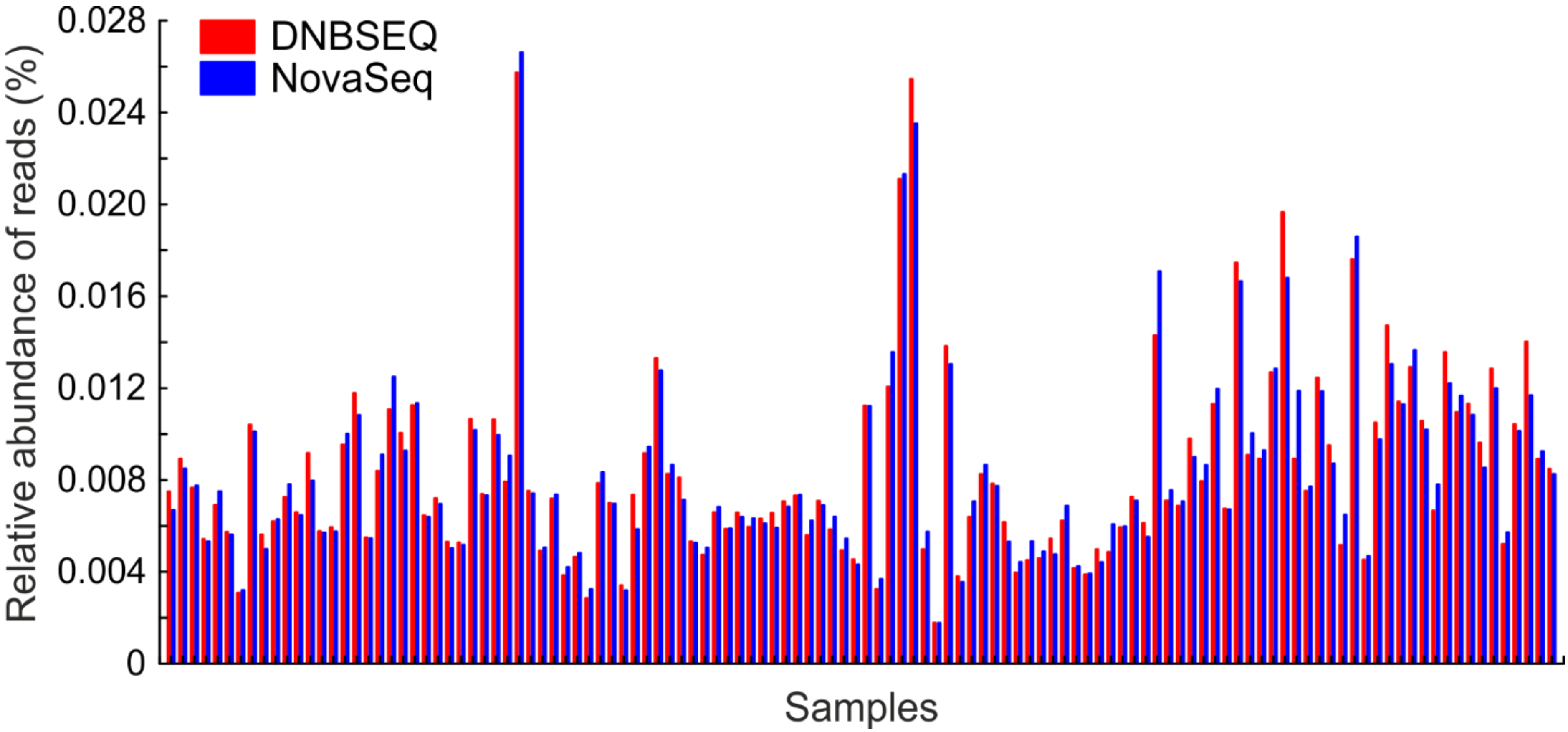

Figure S2. Relative abundance of reads per sample from DNBSEQ and NovaSeq platforms (OTU matrix#1).

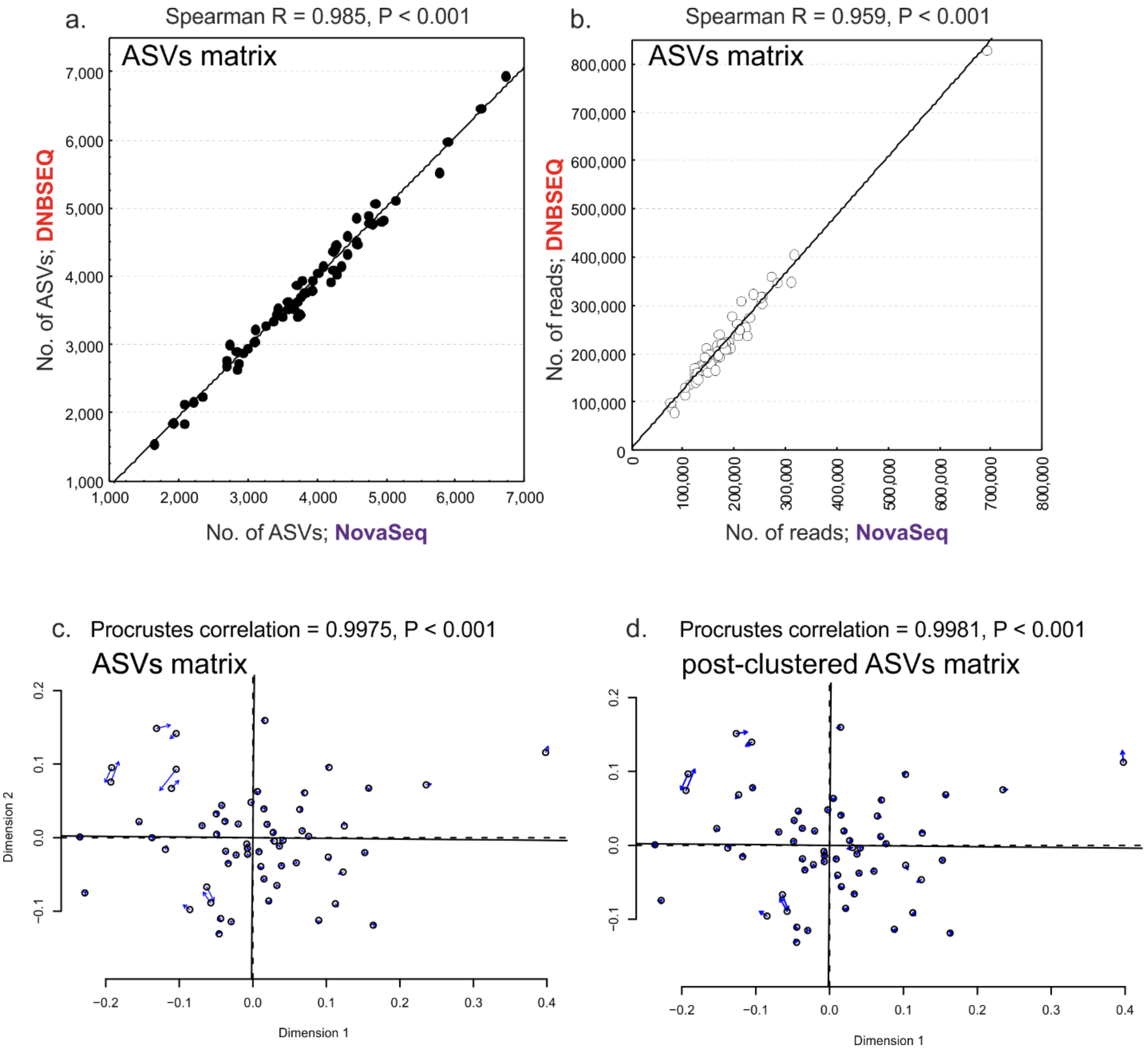

Figure S3. Correlations of number of ASVs (a.) and reads (b.) per sample recorded from DNBSEQ and NovaSeq platforms (Spearman R ≥ 0.95, n = 60, P < 0.001 for all cases). Procrustes test plot (c.) for ASV matrix and (d.) post-clustered ASVs matrix (open circle points in the Procrustes plots denote ordination configuration of NovaSeq data and arrows point to the configuration in DNBSEQ data ordination).

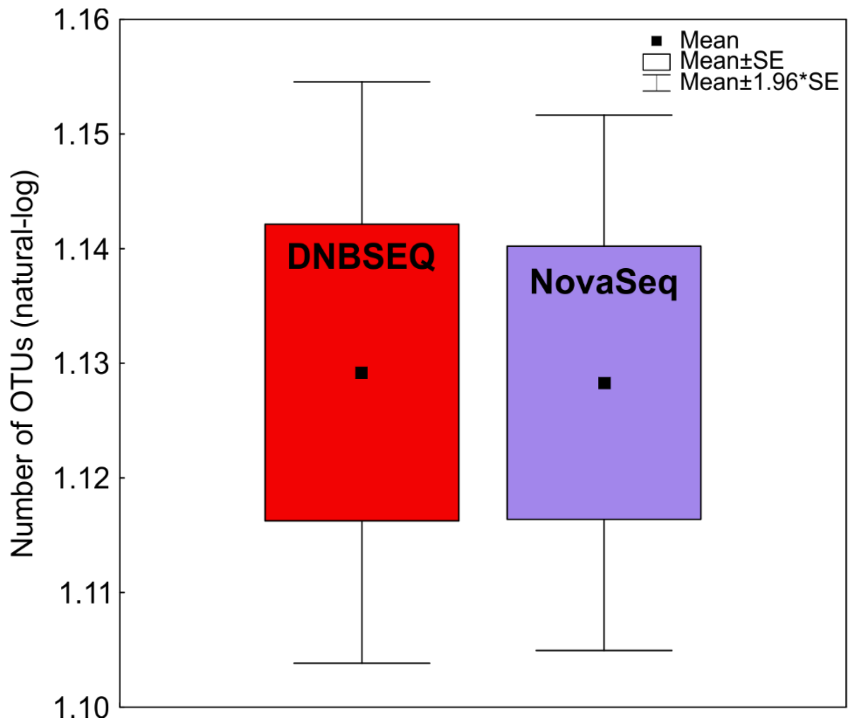

Figure S4. Box-plots for OTU richness between DNBSEQ and NovaSeq data sets including only high identity Metazoan OTUs (≥98%; matrix#5; paired t-test: t = 0.131, df = 119, P = 0.896). ASV data set includes ASVs generated applying DADA2 pipeline on a subset of 60 samples (out of total 120 samples).

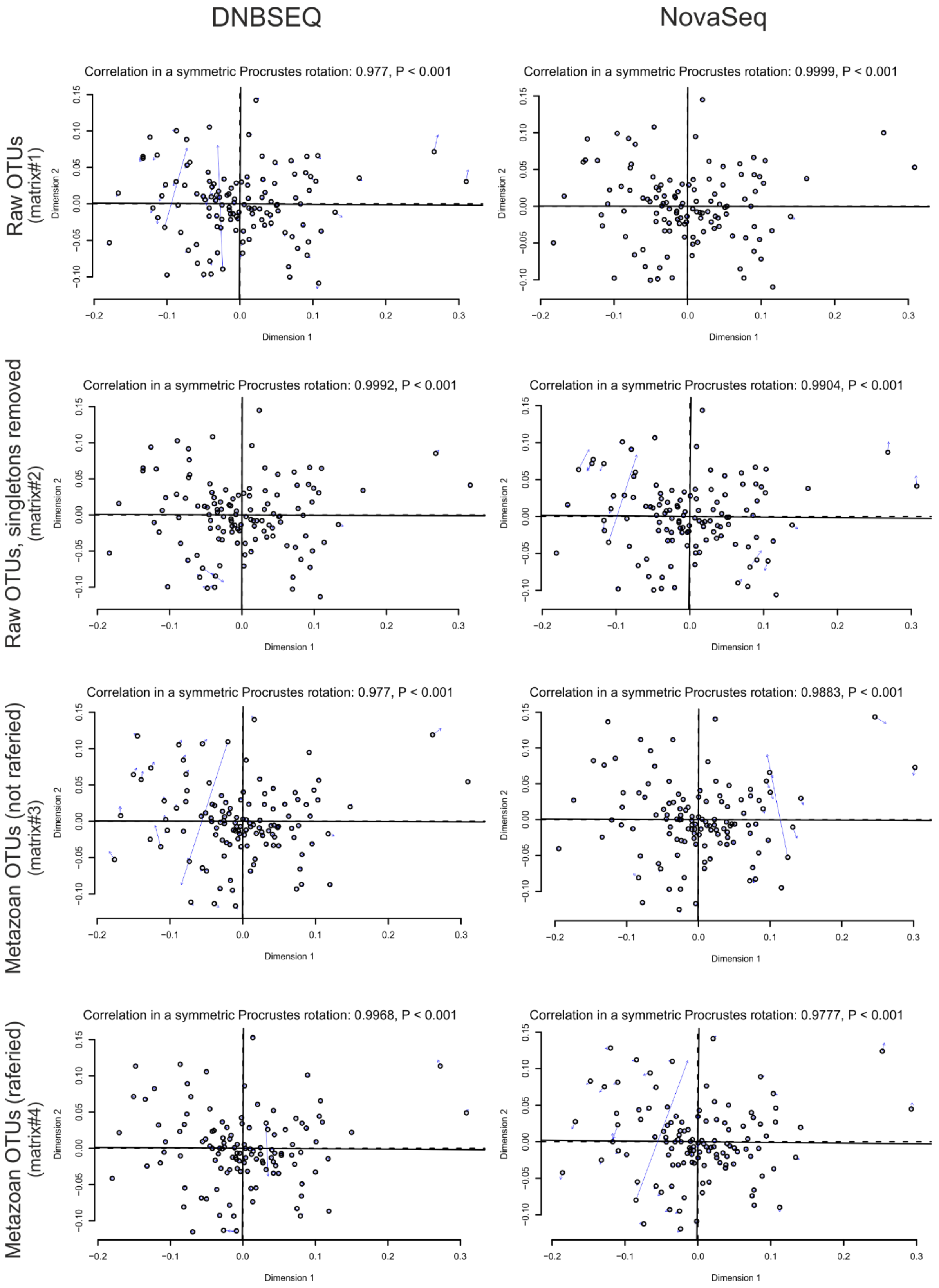

Figure S5. Procrustes test plots for OTU tables from where the tag-switching errors have been corrected according to UNCROSS2 and the uncorrected OTU tables. Open circle points in the Procrustes plots denote ordination configuration of uncorrected data and arrows point to the configuration in tag-switch corrected data ordination.
