# Supplementary material for: Highly comparable metabarcoding results from MGI-Tech and Illumina sequencing platforms
Source: PeerJ. 2021 Sep 30;9:e12254. doi: 10.7717/peerj.12254 (PMC8491618; doi:10.7717/peerj.12254)
Supplement: Supplemental Information 7 [file peerj-09-12254-s007.zip › 1.Legend_for_SupplData.pdf]

OTU_taxonomy.xlsx – OTUs with sequence length 313 +-4 bp supplied with blastn hits against CO1Classifier database v4. **OTU identifiers are uniform across all data matrices.**

matrix1_DNBSEQ.txt – the formed OTU table after clustering filtered reads with 97% similarity threshold with vsearch. Contains data only from **DNBSEQ** platform, corresponds to matrix#1 in the manuscript.

matrix1_DNBSEQ_tagJump_filt.txt – the formed OTU table after clustering filtered reads with 97% similarity threshold with vsearch and applying UNCROSS2 index-switching error correction algorithm. Contains data only from **DNBSEQ** platform.

matrix1_NovaSeq.txt – the formed OTU table after clustering filtered reads with 97% similarity threshold with vsearch. Contains data only from **NovaSeq** platform, corresponds to matrix#1 in the manuscript.

matrix1_NovaSeq_tagJump_filt.txt – the formed OTU table after clustering filtered reads with 97% similarity threshold with vsearch and applying UNCROSS2 index-switching error correction algorithm. Contains data only from **NovaSeq** platform.

matrix2_DNBSEQ.txt – an OTU table derived by removing global singletons from "Full_raw_dataset_with_TAX". Contains data only from **DNBSEQ** platform, corresponds to matrix#2 in the manuscript.

matrix2_DNBSEQ_tagJump_filt.txt – an OTU table derived by removing global singletons from "Full_raw_dataset_with_TAX" and applying UNCROSS2 index-switching error correction algorithm. Contains data only from **DNBSEQ** platform.

matrix2_NovaSeq.txt – an OTU table derived by removing global singletons from "Full_raw_dataset_with_TAX". Contains data only from **NovaSeq** platform, corresponds to matrix#2 in the manuscript.

matrix2_NovaSeq_tagJump_filt.txt – an OTU table derived by removing global singletons from "Full_raw_dataset_with_TAX" and applying UNCROSS2 index-switching error correction algorithm. Contains data only from **NovaSeq** platform.

matrix3_DNBSEQ.txt – an OTU table derived by keeping only potential Metazoan OTUs from matrix#2. See methods section for Metazoan OTU selection. Contains data only from **DNBSEQ** platform, corresponds to matrix#3 in the manuscript.

matrix3_DNBSEQ_tagJump_filt.txt – an OTU table derived by keeping only potential Metazoan OTUs from matrix#2 and applying UNCROSS2 index-switching error correction algorithm. See methods section for Metazoan OTU selection. Contains data only from **DNBSEQ** platform.

matrix3_NovaSeq.txt – an OTU table derived by keeping only potential Metazoan OTUs from matrix#2. See methods section for Metazoan OTU selection. Contains data only from **NovaSeq** platform, corresponds to matrix#3 in the manuscript.

matrix3_NovaSeq_tagJump_filt.txt – an OTU table derived by keeping only potential Metazoan OTUs from matrix#2 and applying UNCROSS2 index-switching error correction algorithm. See methods section for Metazoan OTU selection. Contains data only from **NovaSeq** platform.

matrix4_DNBSEQ.txt – an OTU table derived by subjecting matrix#3 to rarefaction, to a depth of 10,489 reads per sample. Contains data only from **DNBSEQ** platform, corresponds to matrix#4 in the manuscript.

matrix4_DNBSEQ_tagJump_filt.txt – an OTU table derived by subjecting matrix#3 to rarefaction (10,489 reads per sample) and applying UNCROSS2 index-switching error correction algorithm. Contains data only from **DNBSEQ** platform.

matrix4_NovaSeq.txt – an OTU table derived by subjecting matrix#3 to rarefaction, to a depth of 10,489 reads per sample. Contains data only from **NovaSeq** platform, corresponds to matrix#4 in the manuscript.

matrix4_NovaSeq_tagJump_filt.txt – an OTU table derived by subjecting matrix#3 to rarefaction (10,489 reads per sample) and applying UNCROSS2 index-switching error correction algorithm. Contains data only from **NovaSeq** platform.

matrix5_DNBSEQ_HighID_metazoa.txt – an OTU table derived by keeping only abundant and high identity Metazoan OTUs (with relative sequence abundance of ≥ 0.01% and ≥98% sequence similarity to the reference sequences) from matrix#4. Contains data only from **DNBSEQ** platform, corresponds to matrix#5 in the manuscript.

matrix5_NovaSeq_HighID_metazoa.txt – an OTU table derived by keeping only abundant and high identity Metazoan OTUs (with relative sequence abundance of ≥ 0.01% and ≥98% sequence similarity to the reference sequences) from matrix#4. Contains data only from **NovaSeq** platform, corresponds to matrix#5 in the manuscript.

ASV_matrix.xlsx – the formed ASV table with DADA2 pipeline (see file **Bioinformatics.sh**). Contains data from **DNBSEQ** and **NovaSeq** platforms; subset of 60 samples (see Table S1).

post-clustered_ASVs_matrix.txt – post-clustered ASVs matrix by subjecting '**ASV_matrix'** to LULU post-clustering with 97% sequence similarity threshold. Contains data from **DNBSEQ** and **NovaSeq** platforms; subset of 60 samples (see Table S1).

Bioinformatics.sh – codes for all the bioinformatics processing of the sequencing data.

R_codes.pdf – R codes for data analyses.

TagJump_code.R – R codes for calculating index-switches.
